# Supplementary material for: An In Vitro and In Silico Investigation about Monteverdia ilicifolia Activity against Helicobacter pylori
Source: Antibiotics (Basel). 2022 Dec 28;12(1):46. doi: 10.3390/antibiotics12010046 (PMC9854909; doi:10.3390/antibiotics12010046)
Supplement: Supplementary file 1 [file antibiotics-12-00046-s001.zip › antibiotics-2027081-supplementary.docx]

Supplementary File

**Table S1*.*** Urease inhibition results of Monteverdia ilicifolia extracts and semi-purified fractions.

| **Urease Enzyme Inhibition (%±DP)** | **CE1** | **CE2** | **CE3** | **CE4** | **CE5** |
| --- | --- | --- | --- | --- | --- |
| CE | 17.70±1,3 | 19.47±3,96 | 23.02±6,56 | 18.49±5,54 | 27.35±3,06 |
| EAF | 24.28±0,92 | **40.50±3,40** | 32.07±3,55 | 29.36±1,64 | **37.51±3,84** |
| *n*BF | 24.08±1,49 | **37.27±11,97** | 17.19±3,70 | 29.00±1,84 | **47.08±2,85** |
| AQF | 13.33±3,54 | 19.39±4,61 | 26.17±2,64 | 16.01±3,75 | 17.19±2,79 |
| Boric acid | 80 - 90 |  |  |  |  |

CE1= crude extract aqueous; CE2= crude extract ethanol: water 50:50; CE3= crude extract ethanol: water 70:30; CE4= crude extract ethanol: water 96:4; CE5= crude extract acetone: water 7:3; EAF: ethyl acetate fraction; *n*BF: *n-*butanolic fraction; AQF: aqueous fraction.

**Table S2** Compounds identification of nBF5 (acetone: water 7:3 v/v) detected by UHPLC-HRMS negative mode**.**

| **Substance** | **Retention time (min)** | **[M-H]^-^**  ***(m/z)*** | **Main fragments** | **Identification** |
| --- | --- | --- | --- | --- |
| **1** | 3.42 | 387 | 89,119, 179, 272 | n.i. |
| **2** | 3.77 | 387 | 89, 119, 179, 341 | n.i. |
| **3** | 5.30 | 151 | 89, 107, 119, 133 | n.i. |
| **4** | 5.97 | 309 | 103, 113, 173 | n.i. |
| **5** | 6.38 | 105 |  | n.i. |
| **6** | 6.52 | 209 | 85, 99, 111, 129, 159 | n.i. |
| **7** | 6.79 | 267 | 85, 113, 165 | n.i. |
| **8** | 7.12 | 223 | 85, 111, 129, 147, 205 | n.i. |
| **9** | 8.52 | 191 | 87, 111, 129 | n.i. |
| **10** | 11.22 | 203 | 79, 97, 115, 141 | n.i. |
| **11** | 13.43 | 755 | 300 | Quercetin-3-*O*-α-L-rhamnopiranosyl-(1-2)-β-D-glucopiranoside-7-*O*-α-L-rhamonoside |
| **12** | 15.49 | 739 | 284 | Kaempferol-3-galactoside-6-rhamnoside-3-rhamnoside |

*n.i.= non identified compound.

***Table S3.*** Crude extracts and fractions yield from Monteverdia ilicifolia in percentage.

|  | **CE1** | **CE2** | **CE3** | **CE4** | **CE5** |
| --- | --- | --- | --- | --- | --- |
| **EAF** | 35,15 | 19,40 | 23,80 | 28,46 | 24,40 |
| ***n*BF** | 16,77 | 3,37 | 8,04 | 11,13 | 10,56 |
| **AQF** | 31,00 | 72,99 | 52,98 | 32,08 | 52,59 |

(%, w/w; for fractions: %, w/w, related to the crude extract). (CE1= crude extract aqueous; CE2= crude extract ethanol: water 50:50; CE3= crude extract ethanol: water 70:30; CE4= crude extract ethanol: water 96:4; CE5= crude extract acetone: water 7:3; EAF: ethyl acetate fraction; *n*BF: *n*-butanolic fraction; AQF: aqueous fraction).


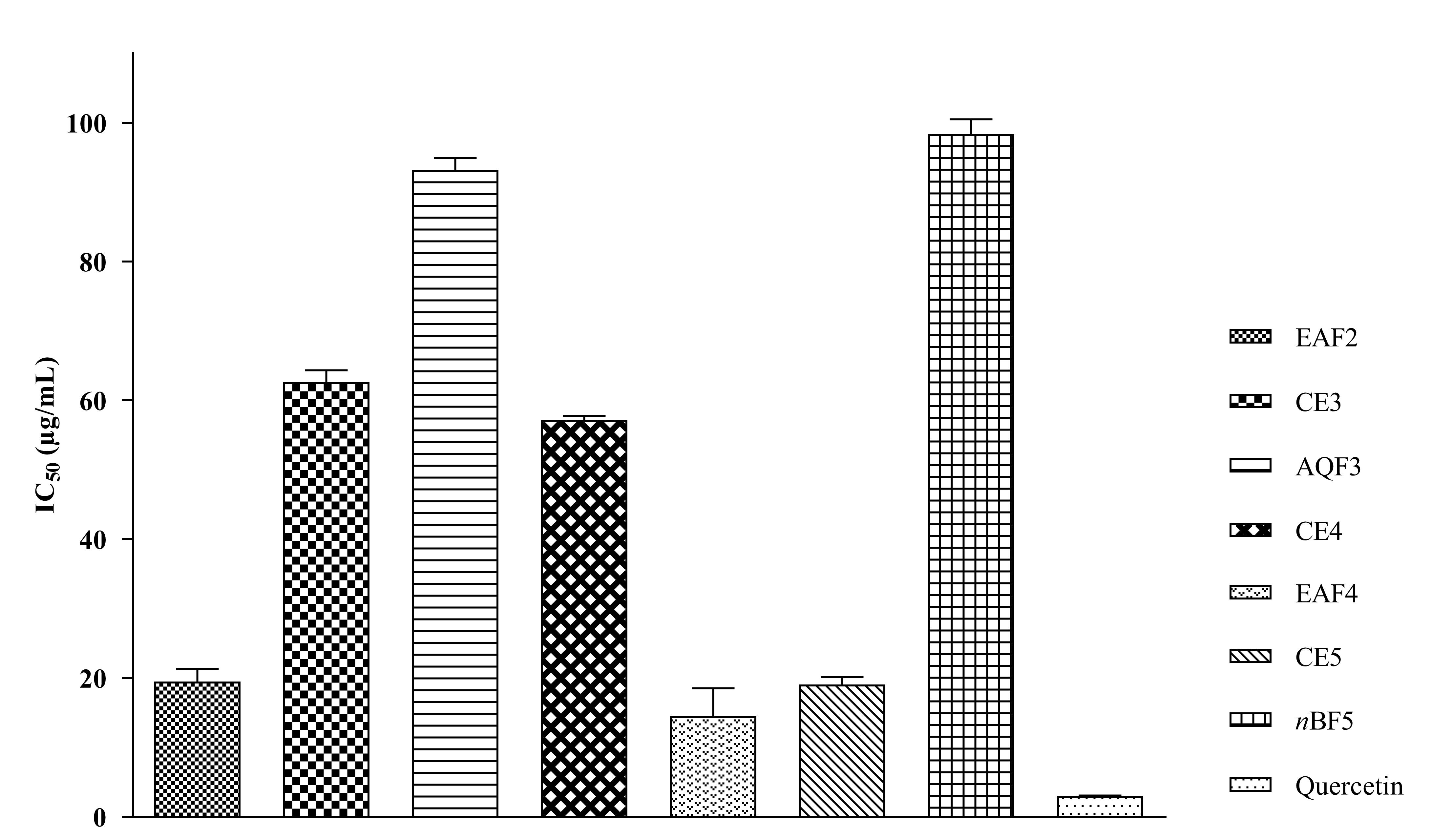


**Figure S1*.*** Antioxidant capacity for the DPPH method in extracts and semi purified fractions of Monteverdia ilicifolia. (CE1= crude extract aqueous; CE2= crude extract ethanol: water 50:50; CE3= crude extract ethanol: water 70:30; CE4= crude extract ethanol: water 96:4; CE5= crude extract acetone: water 7:3; EAF: ethyl acetate fraction; nBF: n-butanolic fraction; AQF: aqueous fraction)*.*


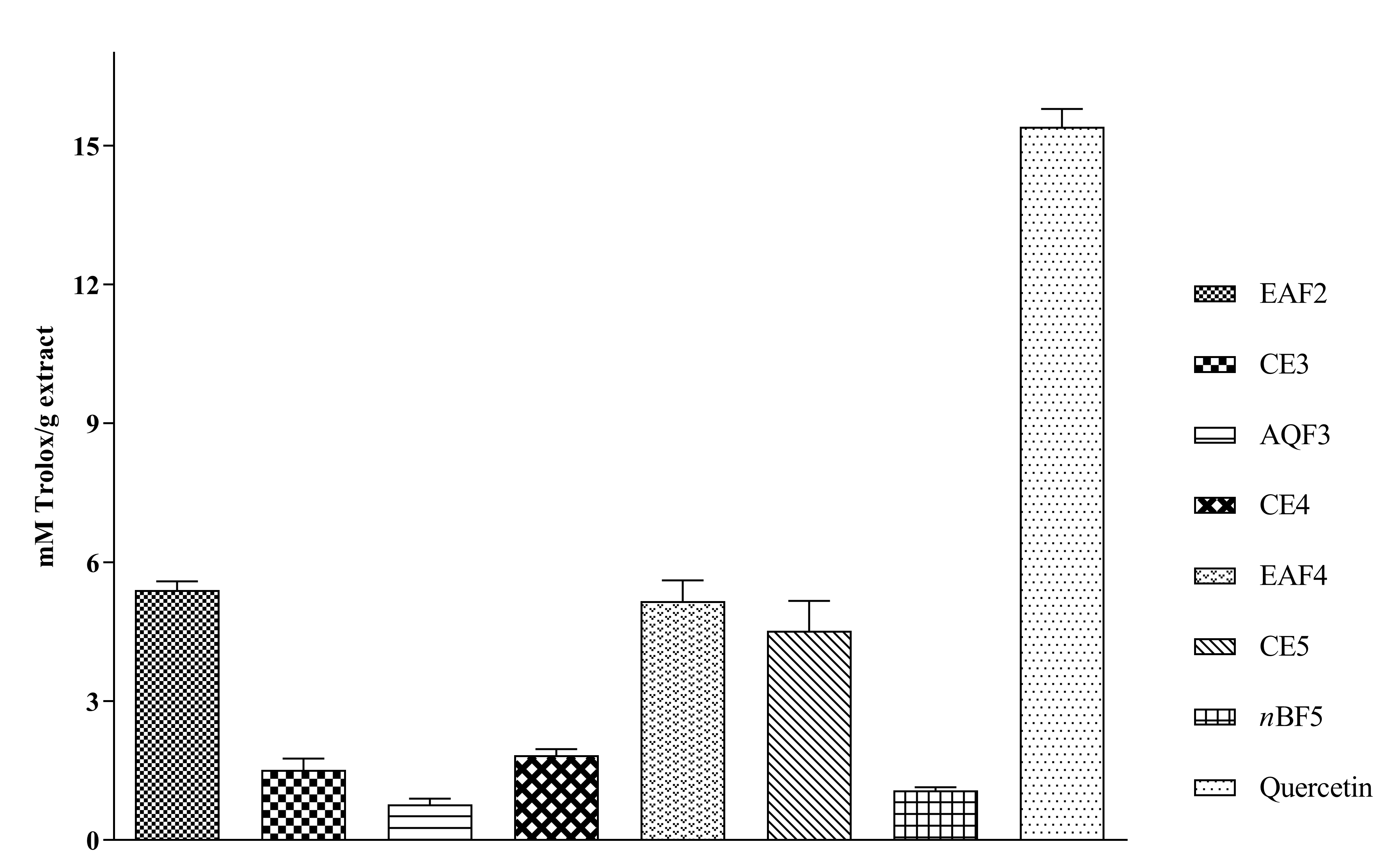


**Figure S2.** Antioxidant capacity for the FRAP method in extracts and semi purified fractions of Monteverdia ilicifolia.(CE1= crude extract aqueous; CE2= crude extract ethanol: water 50:50; CE3= crude extract ethanol: water 70:30; CE4= crude extract ethanol: water 96:4; CE5= crude extract acetone: water 7:3; EAF: ethyl acetate fraction; nBF: n-butanolic fraction; AQF: aqueous fraction).


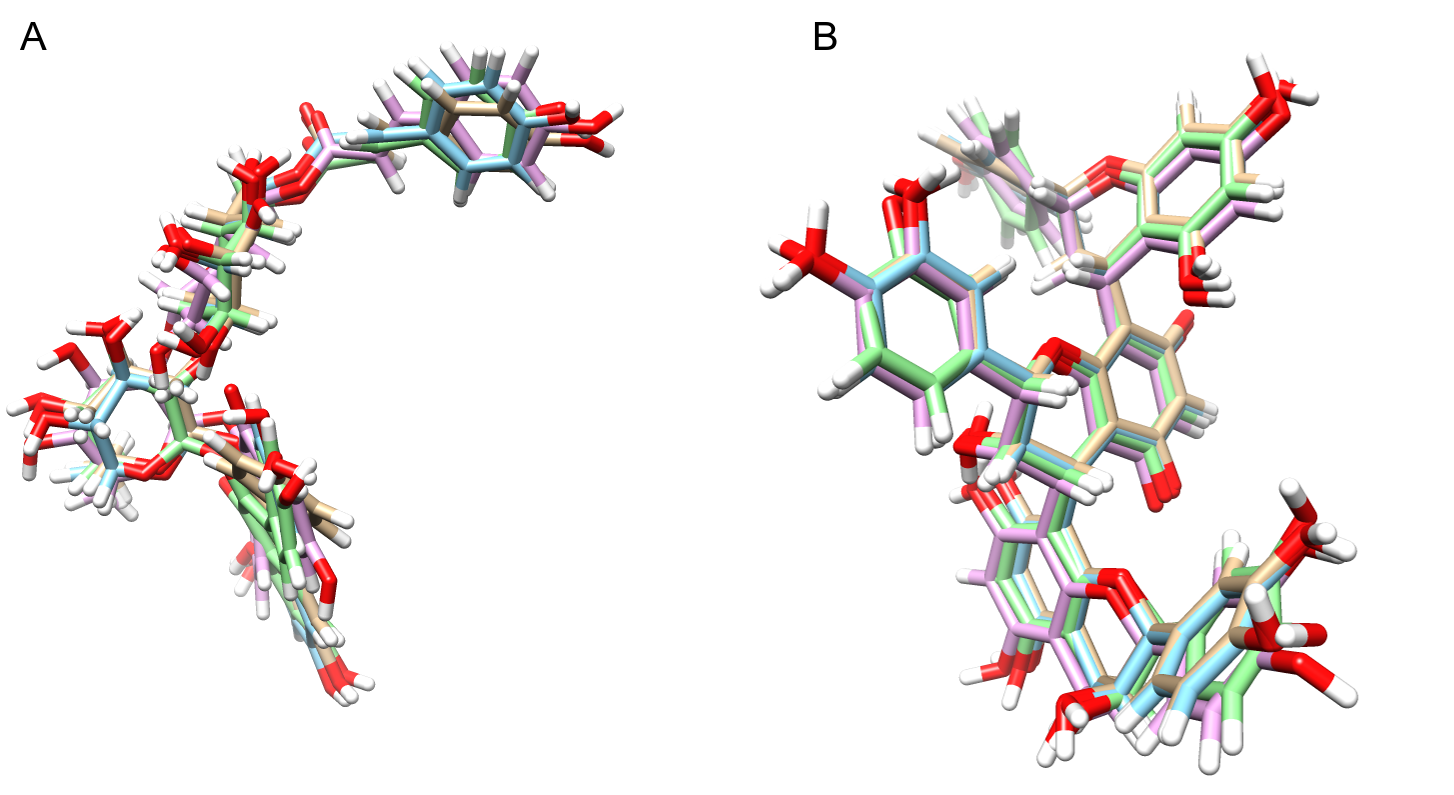


**Figure S3.** Poses found by docking simulations of the ligands Kaempferol-3-galactoside-6-rhamnoside-3-rhamnoside, CID44258967 **(A)** and (epi)afzelechin-(epi)catechin-(epi)catechin, AFZ-CAT-CAT **(B)**, obtained with the Gold program. The repetition of the same conformation for each pose, in all simulations, indicates a pattern of drug-like behaviour.

**Table S4.** Compounds identification of EAF2 and nBF5*.*

| **Compound** | **ID** |
| --- | --- |
| (epi)gallocatechin | CID72277 |
| procyanidin B2 | CID122738 |
| (epi)catechin | CID72276 |
| (epi)afzelechin-(epi)catechin | CID11272988 |
| (epi)afzelechin-(epi)catechin-(epi)catechin | afz-cat-cat-PM_850.sdf |
| (epi)afzelechin-(epi)afzelechin-(epi)catechin | afz-afz-cat-PM_834.sdf |
| kaempferol-galactoside-rhamnoside-rhamnoside | CID23815364 |
| (epi)catechin-(epi)catechin | CID11250133 |
| (epi)afzelechin-(epi)catechin | CID101683293 |
| kaempferol-rhamnopentoside | kaemp-rhamn.sdf |
| Quercetin-3-*O*-α-L-rhamnopiranosyl-(1-2)-β-D-glucopiranoside-7-*O*-α-L-rhamonoside | CID10327769 |
| Kaempferol-3-galactoside-6-rhamnoside-3-rhamnoside | CID23815364  CID11664897  CID21676297  CID44258947  CID134726254  CID10485148  CID44258837  CID44258755  CID6442954  CID14353457  CID131752848  CID44258840  CID14186901  CID5281693  CID44258949  CID44258967 |
